# Identifying the murine mammary cell target of metformin exposure

**DOI:** 10.1038/s42003-019-0439-x

**Published:** 2019-05-20

**Authors:** Mona Shehata, Hyeyeon Kim, Ravi Vellanki, Paul D. Waterhouse, Mathepan Mahendralingam, Alison E. Casey, Marianne Koritzinsky, Rama Khokha

**Affiliations:** Princess Margaret Cancer Centre, University Health Network, Toronto, ON M5G 1L7 Canada

**Keywords:** Breast cancer, Disease model, Target validation

## Abstract

The heterogeneity of breast cancer makes current therapies challenging. Metformin, the anti-diabetic drug, has shown promising anti-cancer activities in epidemiological studies and breast cancer models. Yet, how metformin alters the normal adult breast tissue remains elusive. We demonstrate metformin intake at a clinically relevant dose impacts the hormone receptor positive (HR+) luminal cells in the normal murine mammary gland. Metformin decreases total cell number, progenitor capacity and specifically reduces DNA damage in normal HR+ luminal cells, decreases oxygen consumption rate and increases cell cycle length of luminal cells. HR+ luminal cells demonstrate the lowest levels of mitochondrial respiration and capacity to handle oxidative stress compared to the other fractions, suggesting their intrinsic susceptibility to long-term metformin exposure. Uncovering HR+ luminal cells in the normal mammary gland as the major cell target of metformin exposure could identify patients that would most benefit from repurposing this anti-diabetic drug for cancer prevention/therapy purposes.

## Abstract (web summary)

Mona Shehata et al. study the effects of metformin, an antidiabetic drug with potential as a breast cancer treatment, in the normal mouse mammary gland. They find that metformin targets hormone receptor positive (HR+) luminal cells and decreases the progenitor capacity of these cells when administered at clinically relevant doses.

## Introduction

Breast cancer is one of the leading cancer-related deaths in women. Human breast cancers are very heterogeneous and this poses substantial challenges regarding treatments. Hormone receptor positive (HR+) breast cancers receiving endocrine treatment (tamoxifen) have varied responses and resistance to tamoxifen remains a clinical problem^1^. Since HR+ breast cancers constitute ~70% of all diagnosed cases, there is an important need for improving therapies aimed at these breast cancers.

Repurposing the anti-diabetic drug metformin is at the forefront of targeting human cancers because it is extremely well tolerated in the clinic, and can be given to non-diabetic patients without inducing clinical hypoglycaemia^2^. Epidemiological studies indicate that patients on metformin have lower breast cancer incidence compared to non-metformin users, and other studies suggest that patients taking metformin at the time of breast cancer diagnosis had improved overall survival and/or complete response^3–5^. Metformin reduces the proliferation of multiple breast cancer cell lines via inhibiting Complex I of the electron transport chain^6^, and several studies have shown that metformin delays tumour onset and slows the growth of human xenografts and murine mammary cancer models^7–10^. However, several in vitro and in vivo reports have used non-clinically relevant concentrations that question the validity of repurposing metformin for breast cancer. In addition, there have been no studies investigating how metformin exposure impacts the normal mammary epithelial make-up. The mammary gland is a dynamic tissue composed of two epithelial lineages, luminal and basal, each containing stem- and progenitor-enriched cell fractions. The luminal compartment is further divided into HR+ and HR− populations, while the basal compartment is solely composed of HR− cells, and all have distinct molecular features^11^. Epidemiological and experimental studies collectively suggest that metformin has a potential role in impacting the cell-of-origin of breast cancer.

In this article, we establish the effects of extended metformin treatment on the normal mammary gland. Metformin selectively decreased total cell numbers and progenitor capacity of normal HR+ luminal population, whereas basal and HR− luminal cells were functionally unaffected. Metformin increases the cell cycle length in all luminal cells. Further, HR+ luminal cells demonstrate the lowest levels of mitochondrial respiration, perhaps leaving them more vulnerable to metformin exposure. Metformin also reduces DNA damage levels in the HR+ luminal cells. Thus, we demonstrate that metformin treatment subdues specific mammary cell types and propose that prolonged exposure exerts an anti-cancer effect on HR+ luminal cells.

## Results

### Metformin exposure reduces the normal HR+ luminal population

To study the effects of clinically relevant metformin concentrations^12^ on the normal mammary gland, we continuously treated adult female wild-type mice for prolonged time periods (1 mg/ml; drinking water; 1, 2 and 5 months). Given that adult mammary physiology is hormone-dependent, we documented estrous cycle stages via vaginal smears at time of tissue collection^13^. However, we observed no differences between control and metformin treated mice in estrous stage (Supplementary Fig. 1A).

**Fig. 1:**
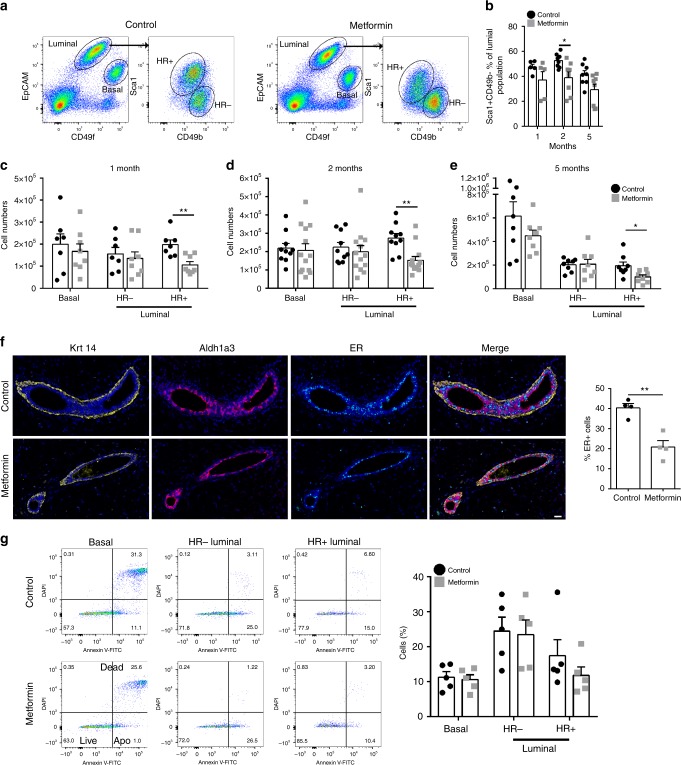
Metformin targets the HR+ luminal compartment. **a** Representative FACS analysis of mammary glands from control vs. 1 mg/ml metformin treated mice, showing luminal (EpCAM+CD49flo) and basal (EpCAMloCD49fhi) subpopulations followed by further segregation of the luminal population into hormone receptor positive (HR+, Sca1+CD49b−) and hormone receptor negative (HR−, SCa1−CD49b+) populations. **b** Summary of HR+/Sca1+CD49b− luminal population flow data in (**a**). HR+ cells from 1, 2 or 5 months treated mice, HR+ cells as a percentage (%) of total luminal cells. *n* = 5–10 (*p = 0.014). Bar chart depicting absolute cell number of epithelial subpopulations from control or 1 mg/ml metformin treated mice after **c** 1 month (**p = 0.0027), **d** 2 months (**p = 0.001) and **e** 5 months (*p = 0.021) of treatment. Values are mean ± SEM. *n* = 7–10. **f** Representative IF images of to delineate the basal (K14+, yellow), HR− luminal (Aldh1a3+, red), and HR+ luminal (ER+, cyan), and DAPI (blue) cells in mammary glands from control or metformin treated mice, scale bar = 25 µm, and quantification of ER+ expression *n* = 4 (**p = 0.0032). **g** Representative FACS analysis with Annexin V-FITC staining. Lower left quadrant represents the live fraction, lower right cells undergoing apoptosis and upper right displaying the dead cells. The bar graph displaying the summary of percentage apoptotic cells from 2-month treated mice. *n* = 5

Isolated mammary cells from 5–10 mice per treatment group were stained with the EpCAM/CD49f/Sca1/CD49b antibody protocol to detect the different mammary subpopulations^14^. In this current study, the mammary glands were processed using the short digestion protocol, resulting in the Sca1+CD49b+ luminal population not being clearly defined, and thus HR+ luminal population most likely included all of the mature HR+ luminal cells and a possible minor subset of the HR+ progenitor population. As shown in Fig. 1a, the basal and HR− luminal populations remain relatively unchanged, whereas the proportion of Sca1+ luminal cells (known to be HR+) was reduced after dosing with metformin (Fig. 1b). The absolute number of HR+ cells dramatically decreased after metformin treatment at all time points compared to controls (Fig. 1c–e). Interestingly, metformin treatment did not noticeably alter the absolute numbers of basal or HR− luminal cells (Fig. 1c–e). Metformin exposure was observed to reduce the number of stromal cells (Supplementary Fig. 1B). The different subpopulations of the mammary gland epithelium can be identified by specific markers in situ, namely Keratins 5 and 14 expression is restricted to the basal compartment, Aldh1a3 has been shown to be highly expressed in the HR− luminal progenitor compartment, and ER (estrogen receptor) is one of the hormone receptors that is strongly expressed in the HR+ luminal compartment. The reduction of HR+ cells in intact tissues from metformin treated mice was confirmed by immunofluorescence staining of mammary glands with markers to visualise the HR+ and HR− luminal cells and basal cells, where we observed the percentage of ER+ cells reduced from 40.39 ± 2.13% in control mice to 20.94 ± 3.14% after metformin treatments (Fig. 1f). The decrease of HR+ luminal cells could arise from increased apoptosis within this population. Annexin V staining demonstrated no difference in either the percentage of apoptotic or dead cells in the basal or HR− luminal compartment after 2 months metformin exposure (Fig. 1g). There was a slight decrease in the HR+ luminal cells after metformin treatment compared to controls, however this was not significant (*p* = 0.56), indicating that metformin treatment does not induce mammary cells to increase the apoptotic program.

### Low OCR of HR+ luminal cells underlie metformin susceptibility

**Fig. 2:**
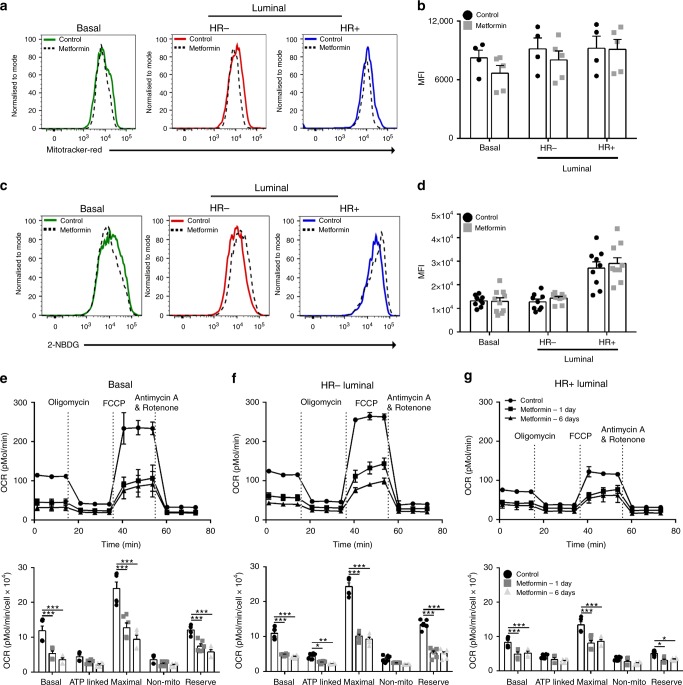
Metformin does not alter metabolism. **a** Mitochondrial mass analysed by flow cytometry for MitoTracker® Red of mammary epithelial cells after 5 months control/metformin treatment. **b** FACS quantification of median fluorescence intensity (MFI) of (**a**) ± SEM. *n* = 4–5. **c** Representative plots of glucose uptake by mammary cells after 5 months treatment determined by 2-NBDG incorporation by flow cytometry. **d** FACS quantification of MFI of (**c**) ± SEM. *n* = 9–10. Oxygen consumption rate was determined using Seahorse XF-96 Metabolic Flux analyser and bar graphs displaying OCR trace (upper panels) the basal, maximal and reserve respiration (lower panels) for **e** basal (***p < 0.001), **f** HR− luminal (***p < 0.001, **p < 0.01, *p < 0.05) and **g** HR+ luminal cells (***p < 0.001, *p < 0.03). *n* = 6

We sought to determine if metformin’s selective effect on the HR+ luminal population was due to a unique metabolic vulnerability of this population. Quantitative digital droplet PCR, a sensitive method for detecting low expressing genes, demonstrated the mammary tissue expresses extremely low levels of Oct3, Mate1 and even lower levels of Oct1, 2 and Mate2, all known transporters of metformin (Supplementary Fig. 2A)^15^. Further analysis of the basal, HR− and HR+ luminal cells for Oct2, Oct3 and Mate1, the major transporters of metformin, also showed extremely low levels of these genes, indicating no difference in expression in any of the mammary epithelial subpopulations after metformin treatment (Supplementary Fig. 2B). We then used the MitoTracker® Red CM-H_2_XRos dye to detect changes in mitochondria activity^16^. Exposure to metformin for either 2 months (Supplementary Fig. 3A–B) or 5 months (Fig. 2a, b) did not alter mitochondrial activity in any of the mammary epithelial cell populations. It has been shown that cells rewire their metabolism to increase glucose uptake in response to metformin^17^. However, no differences existed between control and metformin treated mice in glucose uptake using the non-radioactive fluorescent glucose analogue dye, 2-NBDG at 2 or 5 months (Fig. 2c, d and Supplementary Fig. 3C–D). Therefore, the clinically relevant dose used in this study appears to have no apparent alterations of cellular metabolism in any of the epithelial subsets.

As the total number of HR+ luminal cells was reduced by metformin treatment, we needed to be able to culture HR+ luminal cells in vitro to assess changes to their proliferative potential. A previous report was able to stimulate colony formation in human ERα+ mammary cells with the inclusion of two TGFβ inhibitors^18^. We examined these TGFβ inhibitors in the FAD culture media and observed that these culture conditions allowed the proliferation of HR+ luminal cells (Supplementary Fig. 4). We also observed that the inclusion of Rho kinase inhibitor (Y-27632), which has previously been used to culture mammary basal cells^19,20^, enhanced colony size and numbers (Supplementary Fig. 4).

Next, we asked whether the functional capacity of the mitochondria to handle oxidative stress differs among the mammary populations. We performed the mitochondrial stress test by monitoring changes in the oxygen consumption rate (OCR) of cultured mammary epithelial subpopulations in response to no treatment (baseline respiration), Oligomycin (ATP synthase inhibitor; ATP production), FCCP (uncouple ATP synthesis; maximal respiration), Antimycin A+ Rotenone (Complex III inhibitor and Complex I inhibitor, respectively; non-mitochondrial respiration). Strikingly, the HR+ luminal cells had the lowest baseline OCR compared to the HR− luminal and basal cells, suggesting a low intrinsic dependency on mitochondrial respiration (Fig. 2e–g). Spare respiratory capacity, the difference in maximal and baseline respiration, is the parameter used to measure the capability of cells to respond to metabolic stress. The HR+ luminal cells had the lowest spare respiratory capacity among all the subsets (Fig. 2g), suggesting this population is the most sensitive to oxidative phosphorylation inhibitors.

We next tested whether metformin sensitised these cells to mitochondrial stressors. As was anticipated, we found that in vitro metformin treatment (either 1 or 6 days) profoundly lowered baseline respiration, maximal respiration and spare respiratory capacity in all the mammary epithelial subpopulations (Fig. 2e–g). It is possible that low baseline OCR coupled with the low spare respiratory capacity of the HR+ luminal cells makes these cells metabolically unfit to handle the long-term metformin treatment used in our in vivo studies in contrast to the other subsets (Fig. 2g). Indeed, it has been shown that AML cells with lower reserve capacity show greater sensitivity to oxidative phosphorylation inhibitors^21^.

### Metformin decreases DNA damage levels in HR+ luminal cells

**Fig. 3:**
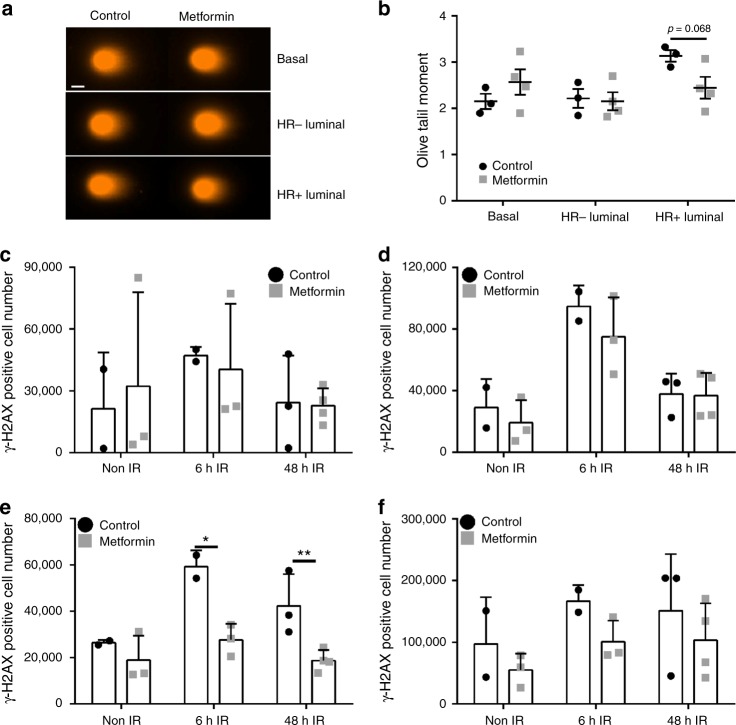
Metformin reduces double stranded DNA breaks in HR+ luminal cells. **a** Representative images from the neutral comet assay of mammary epithelial cells from 2-month treated mice. Scale bar = 5 µm. **b** Olive tail moment showing the geomean ± SEM from at least 100 cells/population from 3–4 independent mice; Students *t*-test. Bar chart depicting γ-H2AX positive absolute cell number from control or 1 mg/ml metformin treated mice in non-irradiated, 6 h post or 48 h post irradiation in **c** basal, **d** HR− luminal, **e** HR+ luminal (*p = 0.021, **p = 0.01) and **f** Stromal cells. Values are mean ± SD. *n* = 2–4; Students *t*-test

Previous studies show that metformin may reduce DNA damage accumulation^22,23^. We thus explored whether normal mammary epithelial cells were less prone to DNA damage after metformin treatment. We performed comet assays on freshly sorted basal, HR− luminal and HR+ luminal cells on control vs. 2-month metformin treated mammary glands (Fig. 3a). Similar levels of DNA double-strand breaks were observed between control and metformin treated HR− luminal cells, while the basal cells showed greater variability in this characteristic after metformin treatment (Fig. 3b). Interestingly, HR+ luminal cells were generally less prone to DNA breaks after metformin exposure compared to control cells (Fig. 3b), suggesting that exposure to metformin may culminate in better DNA repair in the HR+ luminal cells. To test whether metformin may allow HR+ luminal cells to be less prone to DNA breaks, we irradiated mice after 2 months metformin exposure and measured the amount of γ-H2AX in the cells shortly after irradiation (6 h) and after cells had time to recover (48 h). Basal and HR− luminal cells displayed no difference in the number of γ-H2AX positive cells between control or metformin treatments after irradiation, indicating that metformin does not prevent/protect against DNA damage in these cell populations (Fig. 3c, d). Metformin exposure, on the other hand, protects/prevents increased DNA damage in the HR+ luminal cells after irradiation compared to controls (Fig. 3e). The stromal cells followed similar trends to the HR+ luminal compartment with reduced γ-H2AX positive cells, although this was not significant (*p* = 0.41, 0.11, 0.44 respectively, Fig. 3f).

### Metformin treatment lengthens the luminal cell cycle

**Fig. 4:**
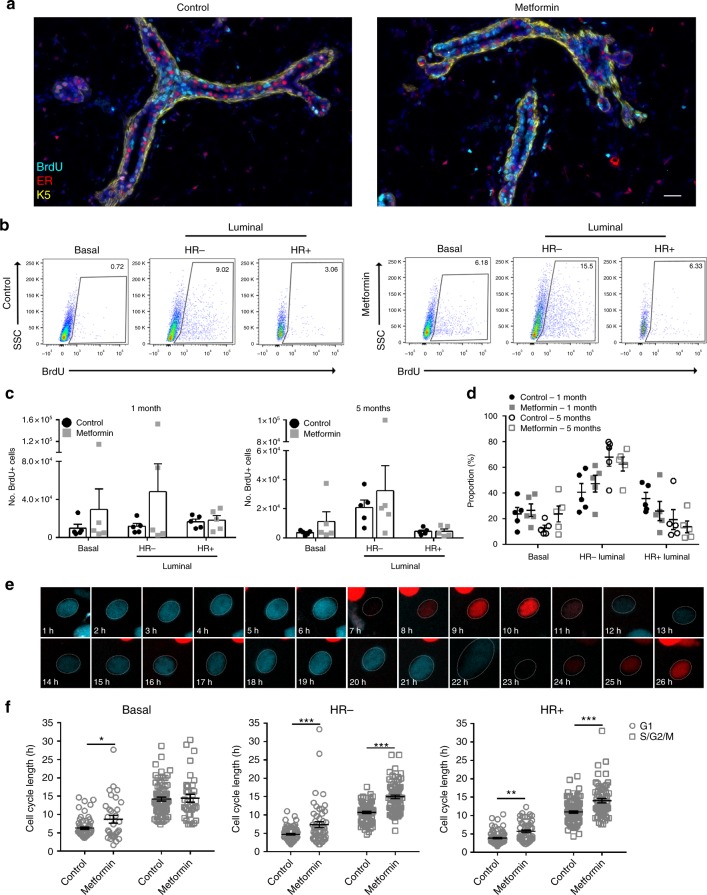
Metformin increases cycle length in the luminal compartment. **a** Representative IF images of mammary glands from mice with continuous exposure of BrdU for the last 7 days of 5 months metformin (or water) treatment. IF images showing BrdU+ (cyan), HR+ luminal (ER+, red), basal (K5+, yellow) and DAPI (blue) cells. Scale bar = 25 µm. **b** FACS plots of BrdU+ cells from basal, HR− and HR+ luminal mammary cells after 5 months treatment. The boxed number indicates % BrdU+ of gated parent population. **c** Absolute numbers of BrdU+ cells from mammary epithelial subsets after 1 or 5 months treatment. Bars depict mean ± SEM. *n* = 5. **d** Dot plot summarising % of BrdU+ basal, HR− and HR+ luminal cells after 1 or 5 months of treatment. *n* = 5. **e** Representative time-lapse images of the cell cycle determined by tracking Fucci2 fluorescence from control vs. metformin treated mammary cells. **f** Quantification of cell cycle length in each of the basal (*p = 0.034), HR− luminal (G1: ***p = 0.0002, S/G2/M: ***p < 0.0001) and HR+ luminal (G1: **p = 0.0019, S/G2/M: ***p < 0.0001) epithelial subpopulations after 1 week in culture, from 50–100 cells from 3 independent mice; 2-way ANOVA

The total cell numbers were reduced only in HR+ luminal cells while metabolic stress was reduced in all populations, suggesting that metformin may impact proliferation in mammary epithelial cells. BrdU was administered continuously via the drinking water for the last 7 days of the treatment period. As shown in Fig. 4a, there were clusters of BrdU+ cells detected in the metformin treated group, in the basal (K5+ER−), HR+ luminal (K5−ER+ cells) and the HR− luminal (K5−ER− cells) cells assessed by immunofluorescent staining. To confirm all mammary epithelial subpopulations underwent cell division, the control and metformin treated glands were analysed by flow cytometry to enumerate BrdU+ cells (Fig. 4b). We observed that there were generally comparable or in some samples more BrdU+ cells after metformin treatment compared to control in the basal and HR− luminal cells (Fig. 4b–d), showing that basal and HR− luminal cell proliferation was unaffected by metformin treatment even after 5 months of treatment. Although the total BrdU+ cells in the HR+ luminal compartment were comparable between control and metformin treated groups, the overall proportion of BrdU+HR+ luminal cells decreased in both 1-month (35.7 ± 4.8% control vs. 26.1 ± 7.5% metformin) and 5-month (19.3 ± 7.7% control vs. 13.7 ± 4.6% metformin) metformin treatment (Fig. 4d).

As metformin is known to induce cell cycle arrest in cancer cells, and we observe that metformin does not entirely compromise the mammary gland’s ability to enter into a proliferative cycle we next determined whether metformin treatment altered aspects of the cell cycle in normal mammary cells. We employed the Fucci2 system, where mCherry-hCdt1 (30/120) is expressed during G1 while mVenus-hGem (1/110) is expressed during S/G2/M phase of the cell cycle^24^. We performed time-lapse microscopy on the different mammary epithelial cells plated in the 2D clonogenic assay from the Fucci2 transgenic mouse and observed the expected transition from red to green fluorescence upon the progression of the cell cycle (Fig. 4e)^25^. Cells were grown in the presence/absence of metformin for 6 days and resulting colonies from basal, HR− luminal and HR+ luminal cells were imaged over 36 h to capture at least one full cell division. Individual cells were tracked to determine time cells spent in the G1 and S/G2/M phases. The average duration of G1 was 4.71 ± 0.21 h in HR− luminal and 3.89 ± 0.18 h in HR+ luminal cells. After metformin treatment, this duration increased by at least a third to 7.36 ± 0.78 h in HR− luminal and 5.72 ± 0.39 h in HR+ luminal cells, while basal cells had a smaller increase in G1 length (6.38 ± 0.32 h to 8.69 ± 1.06 h) (Fig. 4f). Furthermore, the S/G2/M phase also lengthened by almost one third after metformin treatment from 10.69 ± 0.33 h to 14.94 ± 0.52 h in HR− cells and 10.974 ± 0.324 h to 14.039 ± 0.598 h in HR+ luminal cells (Fig. 4f). In contrast, there was no significant difference (*p* = 0.949) between the length of S/G2/M in control vs. metformin treated basal cells.

### Metformin treatment reduces HR+ luminal progenitor activity

**Fig. 5:**
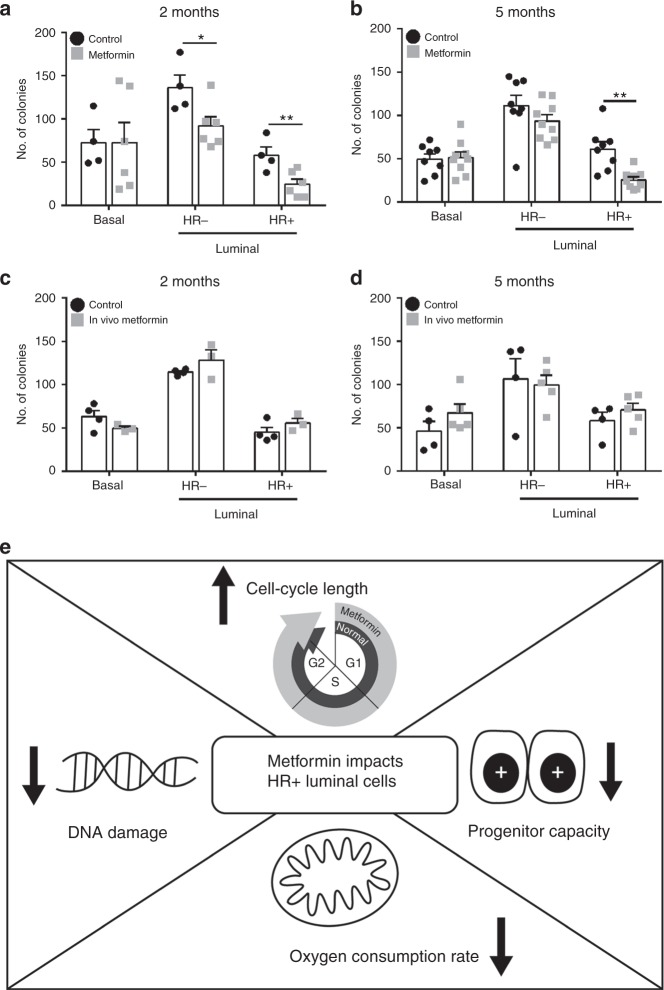
Metformin reduces progenitor activity in HR+ cells. Clonogenic capacity of basal, HR− and HR+ luminal isolated cells after **a** 2-month (*p = 0.037, **p = 0.014) or **b** 5-month control (**p = 0.0012) in vivo/in vitro or metformin in vivo/in vitro treatments. Data presents the mean ± SEM. *n* = 4–9; Students *t*-test. Clonogenic capacity of basal, HR− and HR+ luminal cells isolated after **c** 2-month or **d** 5-month treatments and cultured in the absence of metformin. Data presents the mean ± SEM. *n* = 4–5; Students *t*-test. **e** Proposed model of metformin action on HR+ luminal cells

Progenitor capacity is a measure of lineage maintenance and is typically determined by colony forming assays. To evaluate the progenitor capacity, freshly isolated basal, HR− luminal and HR+ luminal cells from control and metformin treated mice were seeded in 2D clonogenic assays where metformin was included in the culture media for only the metformin exposed mice. The HR− luminal cells displayed reduced progenitor capacity after 2 months of metformin treatment, however this was not significant after 5 months exposure (*p* = 0.208), while the basal cells displayed no change in progenitor capacity after metformin treatment (Fig. 5a). Notably, the HR+ luminal cells displayed a significant decrease (*p* = 0.0012) in progenitor capacity that was sustained over 5 months of metformin exposure (Fig. 5b). To assess whether HR+ cells had permanently reduced progenitor capacity after in vivo metformin exposure, we repeated the 2D clonogenic assays, this time in the absence of metformin in the culture media after in vivo metformin exposure. HR+ luminal cells were able to recover their progenitor activity irrespective of the 2- or 5-month in vivo metformin exposure (Fig. 5c, d) once these cells were no longer exposed to metformin. This suggests that the metformin effects are not permanently altering the progenitor capacity of HR+ luminal cells and once the proliferative pressure is absent the HR+ luminal cells were able to recover normal progenitor capacity. These results demonstrate that metformin effects on progenitor capacity lie almost exclusively within the HR+ compartment in the normal mammary gland.

## Discussion

This study offers critical insight into the action of extended metformin exposure at clinically relevant doses on normal, non-diabetic adult mammary tissue, which has not been investigated to our knowledge. The dose we used of 1 mg/ml in the drinking water translates to plasma concentrations that fall within the range of what is achieved with the 1500 mg/day dose of metformin in humans^12^. Repurposing metformin efficiently for breast cancer therapy or preventative purposes requires an understanding of where it is likely to exert its greatest impact. Metformin’s potential benefit is confounded by epidemiological studies combining breast cancer subtypes into single cohorts^3–5^ or murine tumour models administrated non-physiological doses^8–10^ and not investigating the effects on the normal mammary gland. We show that extended continuous metformin exposure selectively influences the homoeostasis of normal HR+ luminal cells. This is in line with studies that report a delayed tumour onset after lengthy metformin exposure^7,9^, highlighting the potential to impact the tumour initiating cells whereas studies with shorter treatment periods targeting fully formed tumours have shown no effect^26^.

We report that the functionality of the HR+ luminal compartment is most sensitive to metformin treatment. We observe a severe reduction in absolute cell number after extended metformin exposure, while no substantial reduction in cell number was observed in the basal or HR− luminal populations. The effects of metformin may possibly take longer to be observed in these populations, which we reason to be unlikely due to the following evidence. It has previously been shown that ~80% of basal cells, ~90% of HR− luminal cells, and almost 97% of HR+ luminal cells proliferated at least once during extended BrdU exposure^27^ and 5 months metformin exposure would allow 100% of basal, HR− and HR+ luminal cells to have proliferated; only HR+ luminal cells exhibited a reduction. Lineage tracing studies reveal that the majority of the normal homoeostatic adult mammary gland maintenance is via unipotent stem/progenitor cells^20,27–32^, however the occurrence of the rare bipotent stem/progenitor cell has been observed during puberty^33–35^. Stem cell capacity that produces both lineages can be relegated to the developing gland. The adult mice used in our study were confined to a normal homoeostatic environment resulting in only unipotent lineage-restricted maintenance of the basal and luminal populations, where there was no evidence that metformin, at the clinically relevant doses used in this study, displayed any lasting differences in progenitor activity or cell numbers in selected mammary populations. Together, this indicates that extended metformin exposure has minimal effect on the basal and HR− luminal populations.

Metformin induces differentiation in osteoblasts and myoblasts^36,37^, and also promotes neural stem cell self-renewal and differentiation^38,39^. The HR+ luminal population contains mainly terminally differentiated cells^14,40,41^ and metformin further impacts their limited proliferative potential, unlike the basal and HR− luminal compartments that contain more proliferative cells^42,43^. Consistent with this, although metformin extends the cell cycle length in HR− luminal cells their progenitor activity and cell numbers remain uncompromised. Further, given the limited spare respiratory capacity we uncovered in HR+ luminal cells, they are poorly equipped to handle metformin-induced metabolic stress. Since metformin treatment is able to lengthen the luminal mammary cell cycle, extended time spent in S/G2/M cell cycle phases of HR+ cells possibly provides sufficient time for their DNA replication and/or repair resulting in lower baseline levels of DNA breaks. It is likely that as metformin slows down proliferation, it could also protect these HR+ cells from accumulating mutations over time. Our study opens new mechanisms that underlie the selective vulnerability of HR+ luminal cells to metformin, which require further investigation.

Clinical doses of metformin may slightly lower circulating estrogen or cause no change in circulating sex hormone-binding globulin (SHBG) levels in postmenopausal women^44,45^, while premenopausal polycystic ovarian syndrome (PCOS) patients taking clinical doses of metformin showed a modest increase in circulating SHBG and estrogen levels^46^. Numerous women taking metformin have not shown any adverse effects regarding reproduction or lactation, in fact the opposite has been suggested. PCOS women taking metformin reported the regulation of menses and increased ovulation^47^, and metformin did not impact breastfeeding^48^. These clinical studies indicate that metformin effects on circulating hormones are minimal. In the current study, we observed insignificant alterations on the mouse estrous cycle. Further, our investigation shows that only the HR+ luminal compartment had reduced progenitor capacity, in the normal scenario. The HR+ luminal compartment has been shown to be affected by hormonal^14,27^ or microenvironment alterations^49^, and as such, when metformin alters the metabolic state of the mammary gland, the most affected is the HR+ population. These data identify the HR+ luminal compartment to be the most likely to be compromised from metformin therapy, as illustrated in our model (Fig. 5e). In addition to previously reported metabolic factors altered with metformin treatment^50^, our paper demonstrates the importance of understanding the cellular target. HR+ cells have been proposed as the cell-of-origin for ER+ breast cancer^51^ and since ER+ breast cancers comprise the majority of breast cancers diagnosed, repurposing metformin has the potential to broaden prevention/treatment options. Our study highlights the potential of preventing the formation of cancers arising from the HR+ luminal compartment after sustained metformin treatment using current clinical doses.

## Methods

### Mice

Experiments used virgin unsynchronised adult (10+ weeks old) female wildtype C57Bl/6 (Animal Resource Centre), R26p-FUCCI2 (Riken Acc. No. CDB0203T)^24^. For irradiation experiments: Mice were exposed to a single dose of 6 Gy from Cs-137 irradiator (Gammacell® 40 Exactor) and sacrificed at various time-points (i.e. 6 or 48 h) after irradiation for harvesting the mammary glands. All experiments were performed according to guidelines from the Canadian Council for Animal Care and under protocols approved by the Animal Care Committee of the Princess Margaret Cancer Centre, Toronto, Canada.

Estrous staging was determined by cytological characteristics of vaginal smears at time of tissue collection from unsynchronised mice. A vaginal flush was performed with 40 μl of sterile PBS, then spotted onto a microscope slide, adhered for 10 min and visualised under a light microscope. Estrous staging was based on the presence and/or proportion of nucleated epithelial cells, cornified cells and lymphocytes.

### Metformin treatment in vivo

Metformin (Sigma) was dissolved in 200 ml of drinking water to attain the dosage of 1 mg/ml. The water was changed every 2nd day and measured for water intake. Female virgin C57Bl/6 mice were 10 weeks old at the start of metformin treatment and administered metformin continuously for 1, 2 or 5 months, with water only as controls.

### Dissociation of mammary tissue into single cell suspension

The #3 and/or #4 mammary glands (lymph node removed) from female virgin mice were manually minced for 1 min and then enzymatically dissociated for 1.5 h in DMEM/F12 (1:1) supplemented with 2 mg/ml collagenase (Roche) and 200 U/ml hyaluronidase (Sigma). Samples were briefly vortexed every 30 min. The mammary glands were then processed to single cells as previously described^52^. Briefly, after dissociation red blood cells were lysed in ammonium chloride (Stem Cell Technologies) and processed to a single cell suspension by sequential digestion with 0.25% Trypsin (Stem Cell Technologies), 5 mg/ml dispase (Stem Cell Technologies) and 1 mg/ml DNase (Sigma) and filtered through a cell strainer (BD Biosciences).

### Preparation of cells for flow cytometry

Single mammary cells were then incubated with the following primary antibodies (all from BioLegend): CD31-biotin (1:500; clone 390), CD45-biotin (1:500; clone 30-F11), Ter119-biotin (1:500; clone Ter119), EpCAM (1:300; clone G8.8), CD49f (1:400; clone GoH3), CD49b (1:200; HMα2), and Sca1 (1:1000; clone D7). Biotin conjugated antibodies were detected with Streptavidin-eFluor450 (1:500; BioLegend). Cells were then filtered through a 30-μm cell strainer and incubated with 4′,6-diamidino-2-phenylindole (DAPI; Invitrogen) and were analysed by flow cytometry using an LSRII, or sorted on a FACSAria II (Becton Dickinson). The flow-cytometry gating strategy was as previously described^14^. For apoptosis assays: 5 µl Annexin-V FITC (1:100; BioLegend) was added to the standard cell suspension, as above, and incubated at room temperature for 10 min in the dark. Samples were kept on ice until analysed. Flow cytometry data were analysed using FlowJo software (version 10. Tree Star Inc.). For γ-H2AX staining: cells were stained with Zombie UV (BioLegend) instead of DAPI and then fixed in 4% PFA, permeabilized in 0.1% Triton-X-100/PBS before staining with Phospho-Histone H2A.X (Ser139)-FITC (1:200; 20E3, Cell Signaling Technology) on ice for 30 min. Samples were kept on ice until analysed.

The total number of live cells was enumerated following dissociation to single cells. After flow analysis, epithelial cells were calculated by taking the percentage of lineage negative cells multiplied by the total number of all cells. The number of basal/luminal cells is calculated by the percentage of basal or luminal cells multiplied by the total number of epithelial cells. The luminal subpopulations (HR− and HR+ luminal) is again calculated in the same manner, where the percentage of the luminal subpopulations is multiplied by the total number of luminal cells.

### Mitotracker and 2-NBDG

Mitochondrial activity was assessed with MitoTracker® Red CM-H_2_Xros (Molecular Probes), whose accumulation in mitochondria is dependent upon membrane potential.

Glucose uptake per cell was measured using the fluorescent glucose analog 2-[N-(7-nitrobenz-2-oxa-1,3-diazol-4-yl) amino]-2-deoxy-D-glucose (2-NBDG, Molecular Probes).

Cells were dissociated to single cells and stained as described above. Cells were incubated with pre-warmed MitoTracker® Red (500 nM) for 45 min or 2-NBDG (200 nM) for 20 min at 37 °C with 5% CO_2_. All subsequent steps were performed in the dark. Cells were washed in HF, incubated with DAPI and analysed using an LSRII. Data analysis was performed using FlowJo.

### Nucleoside incorporation studies

BrdU (Sigma) was dissolved in PBS to a concentration of 10 mg/ml. For continuous administration of BrdU, mice were given access to water supplemented with 1 mg/ml BrdU ad libitum for 7 days. Bottles containing BrdU water were changed every 2 days and were covered with aluminium foil to prevent light exposure.

BrdU-based incorporation into DNA: After required duration of exposure, mice were culled and mammary glands digested as described above. For intracellular staining, cells were first stained with the indicated surface markers and then fixed with BD Cytofix/Cytoperm Buffer (BD Bioscience) for 20 min at 4 °C, followed by incubation with BD Cytoperm Plus Buffer for 10 min at 4 °C and re-fixed for 5 min at 4 °C. Cells were then treated with DNase I (1 mg/ml, Sigma) in PBS and then immunostained with BrdU-FITC (1:100; clone 3D4).

### Immunostaining and microscopy in tissue sections

Expression of BrdU (1:100; clone BU1/75, Abcam), ER (1:100; clone 6F11, Leica Biosystems), Aldh1a3 (1:200; clone HPA046271, Sigma), Krt 5 (1:500; polyclonal, Abcam), and/or Krt 14 (1:1000; polyclonal, Covance) in intact mammary glands was done by fixing freshly isolated glands in 4% PFA overnight and processing the tissue into paraffin. Tissue blocks were sectioned at 4 µm, deparaffinised in xylene, gradually rehydrated in descending concentrations of ethanol, and subsequently treated in Borg Decloaker antigen retrieval solution (pH 6) for 30 min at 121 °C and 10 s at 90 °C using a Decloaking chamber (Biocare Medical). The samples were preblocked in PBS with 1% BSA and 0.1% Tween 20, and then incubated with primary antibodies overnight at 4 °C. The secondary antibodies were goat anti-mouse AF647, goat anti-rabbit Cy3, goat anti-rat AF488 and/or goat anti-chicken AF488 (Invitrogen). No primary antibody was used as a control. Sections were mounted with ProLong Gold antifade with DAPI. Tissue sections were imaged using the Olympus BX50 microscope (Olympus).

### Mammary CFC assay

For CFC assays 500 sorted basal, HR− luminal cells, or 1000 HR+ luminal cells were seeded in complete FAD media (3:1 DMEM/F12 + 1.8 × 10^−4^ M adenine + 1.8 × 10^−3^ M calcium) + 10% FBS (Gibco) + 0.5 μg/ml hydrocortisone (Sigma) + 10^−10^ M cholera toxin (Sigma) + 10 ng/ml epidermal growth factor (EGF, Stem Cell Technologies) + 5 μg/ml insulin (Gibco) + 10 μM Y-7632 (Sigma) + 50 μg/ml gentamicin (Sigma) in the presence of irradiated feeders and cultured for 7 days at 37 °C in a hypoxic (5% O_2_ and 5% CO_2_) incubator. For HR+ luminal cells, two TGFβ inhibitors (25 µM RepSox and 10 µM SB431542, Sigma-Aldrich) were included added to the culture media after 2 days. For metformin treatment in vitro, 1 mM metformin was added to the culture media from the beginning of the assay. After 7 days, the colonies were fixed with acetone/methanol (1:1), stained with Giemsa (Fisher Scientific), and enumerated under a microscope.

### Seahorse

OCR measured using a Seahorse XF96 Extracellular Flux Bioanalyzer (Seahorse Bioscience). Cells seeded at appropriate density in specialised Seahorse tissue culture plates (4 wells/condition) for 6 days. Metformin was added to the cells either at the beginning of the assay, or 1 day prior to analysis. On the day of analysis, cells were washed with 1× PBS and then with sodium bicarbonate free DMEM/F12 (pH 7.4) + 1% FBS. Cells were then incubated in sodium bicarbonate-free complete DMEM/F12 media for 1 h. Baseline OCR was determined. Mitochondrial stress test was performed to gather information about mitochondria/glycolytic function of mammary epithelial cells in response to metformin exposure using Oligomycin (ATP-synthase inhibitor), FCCP (mitochondria uncoupler), Rotenone (complex I inhibitor) and Antimycin A (cytochrome C reductase inhibitor) (all from Sigma) so a profile of ATP-lined, maximal and non-mitochondrial respiration could be determined. OCR was normalised for the number of cells in each well using the seahorse software. Cell numbers were quantified using CyQuant (Life Technologies) according to manufacturer's instructions on the seahorse plate once seahorse measurements were completed. Four biological replicates were performed and the measurements were combined for analysis.

### Live cell imaging

Sorted basal, HR− and HR+ luminal R26p-Fucci2 cells were grown on Eppendorf Cell Imaging 24 well plates with cover glass (Eppendorf). After 4 days, cells were imaged in phenol red-free complete FAD media with 1 mM metformin or water for the control group, in a 5% CO_2_/37 °C incubation chamber on an AxioObserver microscope (Zeiss) with a 20×/0.8 DIC Plan-Apochromat objective (Zeiss) at 20 min intervals for 36 h. Green (470 nm) and red (545 nm) signals were acquired in dual camera mode. The 2D images were processed by Zen 2.3 software (Blue edition, Zeiss). All images were analysed by Image J (FIJI-64 bit) software. S/G2/M phase was measured as the time between first and last frame with green fluorescence. G1 was measured as the time between the first frame without fluorescence (after cell division) and the last frame with red fluorescence. This includes a short interval of S phase in which the cells were both green and red.

### Comet assay

Freshly sorted basal, HR−, and HR+ luminal cells were mixed with 0.7% low melting point agarose gel (molten and at 37 °C) at 1:10 ratio. Each mixture was laid on a glass slide pre-coated with 1% normal melting point agarose and immediately covered with an 18 × 18 mm coverslip. The coverslips were removed after gel solidification at 4 °C, and the samples were then processed according to the manufacturer’s recommendation (Trevigen). Briefly, slides were lysed at 4 °C O/N and then immersed in 1× Neutral Electrophoresis buffer for 30 min. The slides were transferred to the CometAssay® electrophoresis unit filled with chilled 1× Neutral Electrophoresis buffer and electrophoresed at 21 V for 7 min at 4 °C. The slides were then fixed in DNA Precipitation Solution followed by 70% ethanol for 30 min each at room temperature. The slides were dried and stained with SYBR® Gold gel staining solution (Thermo Fisher). The Olive-tail moment was quantified using Komet Software (Andor Technology) and each data point indicates a geometric mean of Olive tail moment obtained from at least a 100 comets per mouse.

### Droplet digital PCR

RNA from freshly sorted basal, HR− and HR+ luminal cells, from mice subjected to control or metformin, was extracted using the RNeasy micro kit (Qiagen) as per manufacturer's instructions. cDNA was generated using the ProtoScript II First Strand cDNA Synthesis Kit (New England Biolabs). ddPCR was performed on a QX200 droplet digital PCR system (BioRad) with Oct2 (F: CGCTGGTCGTATTTGCTGTG; R: AAGCTCTGCCTCTGGTCTCA), Oct3 (F: GCCCGGAGCTCTCTTAATCC; R: CTCAGCCACGGTATCCCTTC), Mate1 (F: TTCCTAGAGGAGCTGCGGG; R: CGTATGTCTGGGAGATGAGCG) primers. The PCRs were performed in a total volume of 20 µl containing 10 µl 2× ddPCR Supermix, primers (9 nM forward and 9 nM reverse), 5 ng DNA and water. The reaction mixtures were partitioned into an emulsion of approximately 20,000 droplets in oil using a QX200 droplet generator. The droplets were then transferred to a 96-well PCR plate, heat sealed and amplifications were performed using the following conditions: 10 min hold at 95 °C, 40 cycles of 95 °C for 15 s then 60 °C for 60 s. Once completed, the plate was loaded onto a BioRad QX200 droplet reader and read using BioRad QuantaSoft™ software.

### Statistics and reproducibility

Statistical analysis. Data are presented as the mean ± SEM of 3–10 independent experiments, except for Fig. 3c–e which is presented as mean ± SD of 2–4 independent experiments. Comparisons between multiple groups were analysed using analysis of variance (ANOVA) followed by a multiple comparisons test, or a Student’s *t*-test, indicated in the figure legends. GraphPad Prism v. 6.0. Statistical significance is indicated as follows: *p < 0.05, **p < 0.01 and ***p < 0.001.

### Reporting summary

Further information on research design is available in the Nature Research Reporting Summary linked to this article.

## Supplementary information

Supplementary Information

Reporting Summary

## Data Availability

The data sets generated during the current study are available to the reader upon request from the corresponding author.
