# Supplementary material for: Identifying the murine mammary cell target of metformin exposure
Source: Commun Biol. 2019 May 20;2:192. doi: 10.1038/s42003-019-0439-x (PMC6527562; doi:10.1038/s42003-019-0439-x)
Supplement: Supplementary file 1 — Supplementary Information [file 42003_2019_439_MOESM1_ESM.pdf]

A

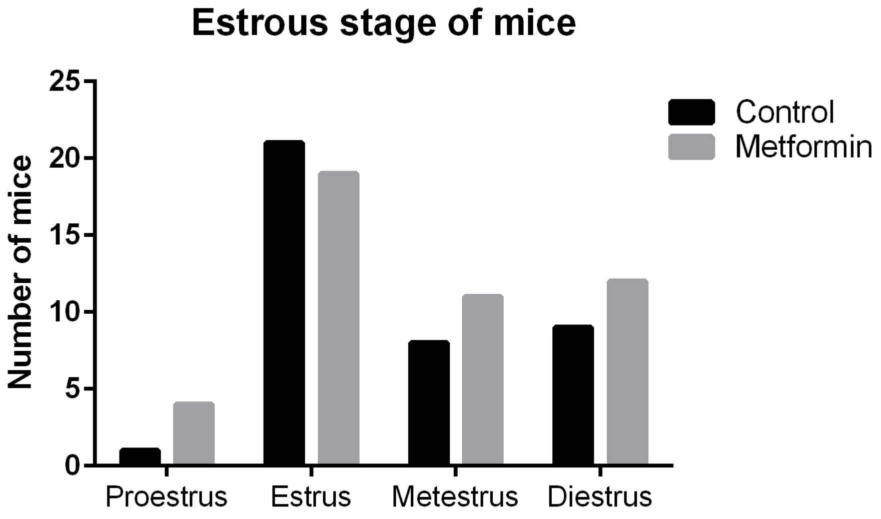

B

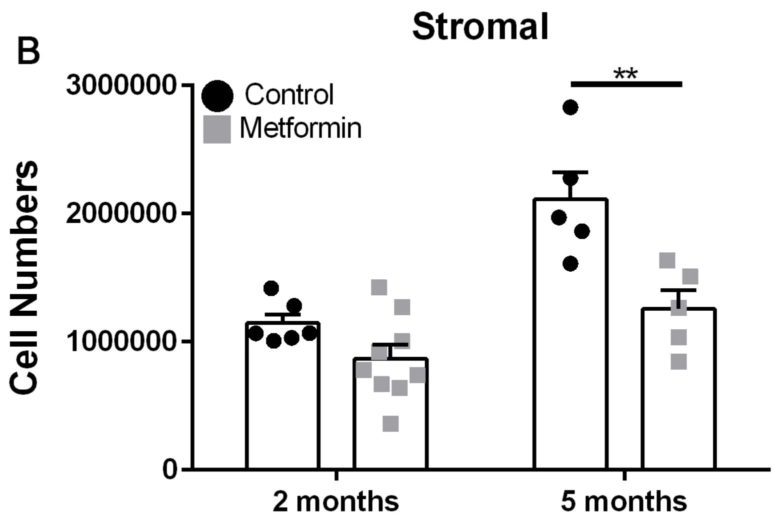

Supplementary Figure 1. Estrous stage and stromal cell number after Metformin treatment. A) Bar chart depicting the number of mice within the different estrous stages. B) Bar chart depicting absolute cell number of stromal cells from control or 1mg/ml metformin treated mice after 2 months and 5 months of treatment (**p<0.01). Mean ± SEM. n=5-9; student's t-test.

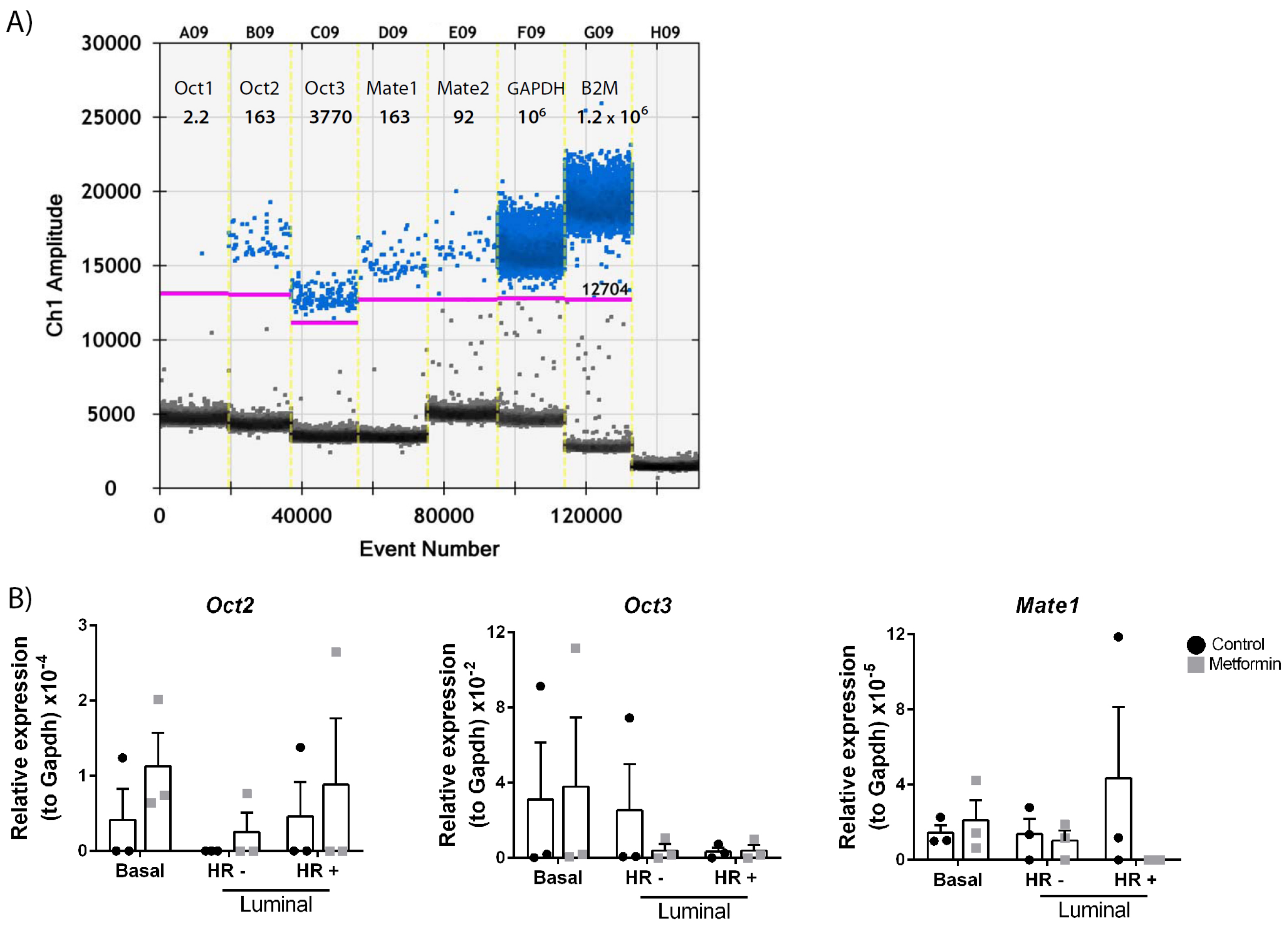

Supplementary Figure 2. Expression of metabolic transporters in mammary epithelial subpopulations after 2 months metformin treatment.

A) ddPCR of Oct1, Oct2, Oct3, Mate1, Mate2 and two housekeeping genes (Gapdh and β2M) expression in total mammary gland tissue. B) Digital droplet PCR analysis of Oct1, Oct2 and Mate1 in basal, HR- luminal and HR+ luminal cells from 2 month treated mice. Mean ± SEM. n=3.

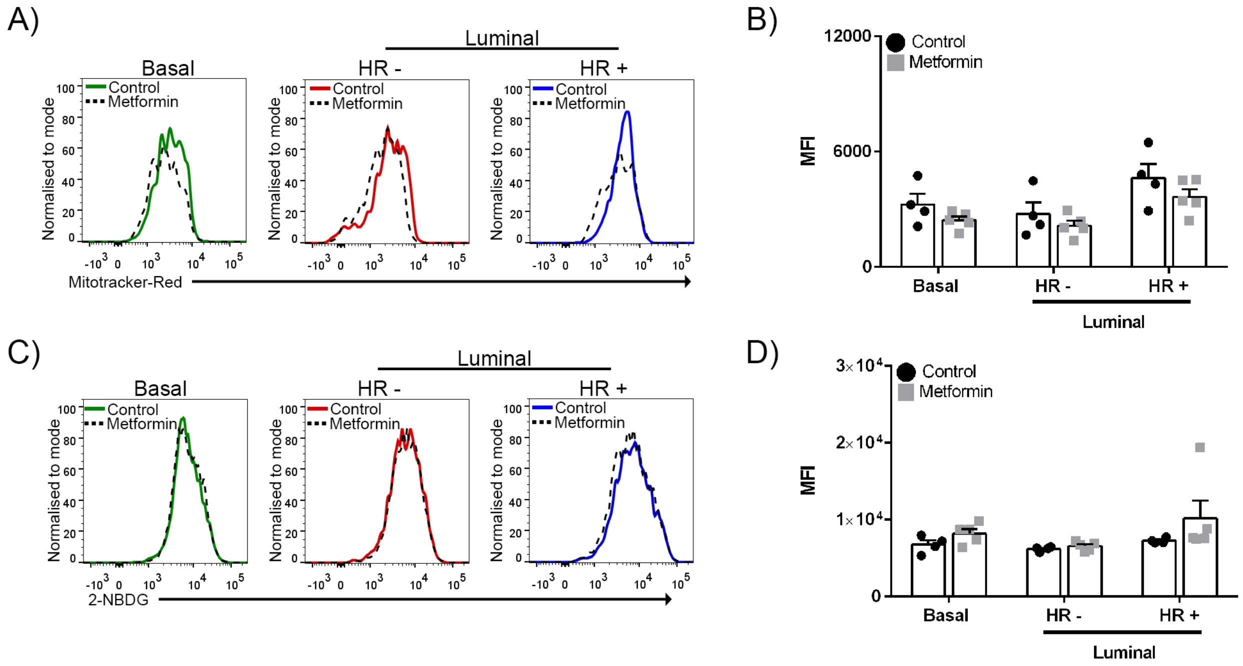

Supplementary Figure 3. Mitochondrial mass and glucose uptake after 2 months metformin treatment.
A) Mitochondrial mass analysed by flow cytometry for MitoTracker® Red of mammary epithelial cells after 2 months control/metformin treatment. B) FACS quantification of median fluorescence intensity (MFI) of Supplementary Figure 3A ± SEM. n=4-5. C) Representative plots of glucose uptake by mammary cells after 2 months treatment determined by 2-NBDG incorporation by flow cytometry. D) FACS quantification of MFI of Supplementary 3C ± SEM. n=4-5.

A)

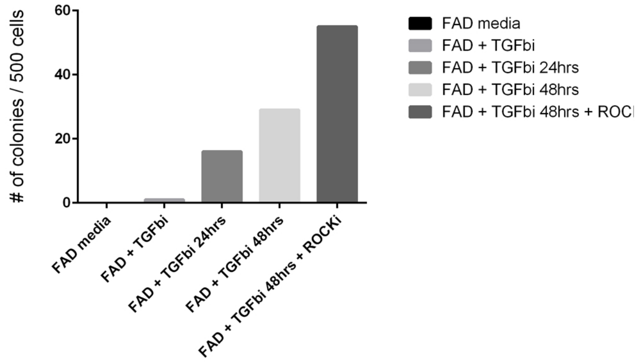

B)

FAD + TGFβi 48hrs

FAD + TGFβi 48hrs + ROCKi

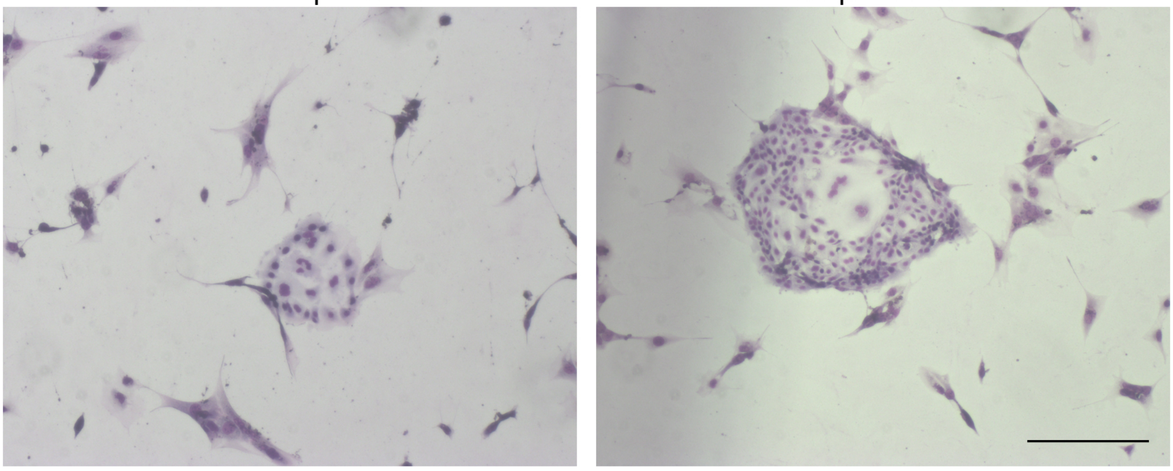

Supplementary Figure 4. HR+ luminal culture.

A) HR+ luminal cell clonogenic capacity after the addition of TGFβ inhibitors (RepSox and SB 431542) or TGFβ inhibitors and ROCK inhibitors after plated on feeders in the presence of complete FAB media. B) Representative bright field images of colonies from HR+ luminal cells. Scale bar = 250 μm.
