# Supplementary material for: Identifying the murine mammary cell target of metformin exposure
Source: Commun Biol. 2019 May 20;2:192. doi: 10.1038/s42003-019-0439-x (PMC6527562; doi:10.1038/s42003-019-0439-x)
Supplement: Supplementary file 2 — Reporting Summary [file 42003_2019_439_MOESM2_ESM.pdf]

nature research

Corresponding author(s): Rama Khokha

Last updated by author(s): Apr 16, 2019

Reporting Summary

Nature Research wishes to improve the reproducibility of the work that we publish. This form provides structure for consistency and transparency in reporting. For further information on Nature Research policies, see Authors & Referees and the Editorial Policy Checklist.

Statistics

For all statistical analyses, confirm that the following items are present in the figure legend, table legend, main text, or Methods section.

n/a	Confirmed	
☐	☒	The exact sample size (n) for each experimental group/condition, given as a discrete number and unit of measurement
☒	☐	A statement on whether measurements were taken from distinct samples or whether the same sample was measured repeatedly
☐	☒	The statistical test(s) used AND whether they are one- or two-sided<br>*Only common tests should be described solely by name; describe more complex techniques in the Methods section.*
☒	☐	A description of all covariates tested
☒	☐	A description of any assumptions or corrections, such as tests of normality and adjustment for multiple comparisons
☐	☒	A full description of the statistical parameters including central tendency (e.g. means) or other basic estimates (e.g. regression coefficient) AND variation (e.g. standard deviation) or associated estimates of uncertainty (e.g. confidence intervals)
☒	☐	For null hypothesis testing, the test statistic (e.g. F, t, r) with confidence intervals, effect sizes, degrees of freedom and P value noted<br>*Give P values as exact values whenever suitable.*
☒	☐	For Bayesian analysis, information on the choice of priors and Markov chain Monte Carlo settings
☒	☐	For hierarchical and complex designs, identification of the appropriate level for tests and full reporting of outcomes
☒	☐	Estimates of effect sizes (e.g. Cohen's d, Pearson's r), indicating how they were calculated

Our web collection on statistics for biologists contains articles on many of the points above.

Software and code

Policy information about availability of computer code

Data collection	All software used is commercially or freely available. Flow cytometry data collected using FACSDiva (v8). Live cell imaging collection with Zeiss (Blue v2.3) and Seahorse datacollection with Wave (v2.0).
Data analysis	All software used is commercially or freely available. Statistical data analysis was performed with Prism (GraphPad, v6). Flow cytometry data analyses using FlowJo (v10). Image analysis with Zeiss (Blue v2.3) and Image J (FIJI-64 bit). Seahorse analysis with Wave (version 2.0).

For manuscripts utilizing custom algorithms or software that are central to the research but not yet described in published literature, software must be made available to editors/reviewers. We strongly encourage code deposition in a community repository (e.g. GitHub). See the Nature Research guidelines for submitting code & software for further information.

Data

Policy information about availability of data

All manuscripts must include a data availability statement. This statement should provide the following information, where applicable:

- Accession codes, unique identifiers, or web links for publicly available datasets
- A list of figures that have associated raw data
- A description of any restrictions on data availability

The datasets generated during and/or analysed during the current study are available from the corresponding author on request.

Field-specific reporting

Please select the one below that is the best fit for your research. If you are not sure, read the appropriate sections before making your selection.

☒ Life sciences ☐ Behavioural & social sciences ☐ Ecological, evolutionary & environmental sciences

Life sciences study design

All studies must disclose on these points even when the disclosure is negative.

Sample size	No statistical method was used to predetermine sample size.
Data exclusions	No other data was excluded.
Replication	All experimental findings from the multiple independent experiments were reliably reproduced.
Randomization	No randomization was performed.
Blinding	No blinding was used. The experiments were quantified and where relevant appropriate statistical tests performed.

Reporting for specific materials, systems and methods

We require information from authors about some types of materials, experimental systems and methods used in many studies. Here, indicate whether each material, system or method listed is relevant to your study. If you are not sure if a list item applies to your research, read the appropriate section before selecting a response.

Materials & experimental systems

n/a	Involved in the study	
☐	☒	Antibodies
☒	☐	Eukaryotic cell lines
☒	☐	Palaeontology
☐	☒	Animals and other organisms
☒	☐	Human research participants
☒	☐	Clinical data

Methods

n/a	Involved in the study	
☒	☐	ChIP-seq
☐	☒	Flow cytometry
☒	☐	MRI-based neuroimaging

Antibodies

Antibodies used	This information is provided in the methods section.<br>Antibodies used for flow cytometry were:<br>CD31-biotin (Clone 390, 102404, Biolegend), CD45-Biotin (Clone 30-F11, 103103, Biolegend), Ter119-biotin (clone Ter119, 13-5921-81, eBioscience), EpCAM (Clone G8.8, 118211 or 118217, BioLegend), CD49f (clone GoH3, 313621, BioLegend), CD49b (HMα2, 103515 or 103506 BioLegend), and Sca1 (clone D7, 108131, BioLegend), BrdU-FITC (Clone 3D4, 364103, Biolegend), Phospho-Histone H2A.X-FITC (20E3, 9718, Cell Signaling Technology), Annexin-V-FITC (Annexin A5, 640905, Biolgened), MitoTracker Red CM-H2Xros (M7513, Molecular Probes). Biotin conjugated antibodies were detected with Streptavidin-eFluor450 (48-4317-82, eBioscience).<br>Antibodies for IF staining:<br>rat anti-BrdU (clone BU1/75, Abcam, ab6326), mouse anti-ERα (6F11, Novocastra, NCL-ER-6F11), rabbit anti-Keratin 5 (polyclonal, Abcam, ab53121), rabbit anti-Aldh1a3 (clone HPA046271, Sigma), chicken anti-Keratin 14 (polyclonal, Covance). The secondary antibodies were goat anti-mouse AF647 (115-605-166, Jackson Labs), goat anti-rabbit Cy3 (111-165-003, Jackson Labs), goat anti-rat AF488 (A-11006, Invitrogen) and/or goat anti-chicken AF488 (A11039, Invitrogen).
Validation	All antibodies had been validated in previous publications .

Animals and other organisms

Policy information about studies involving animals; ARRIVE guidelines recommended for reporting animal research

Laboratory animals	Experiments used virgin adult (10+ week old) female wildtype C57Bl/6 (OCI) and R26p-FUCCI2 (Riken Acc. No. CDB0203T) were used in this study and have been previously described. For all experiments, adult female virgin mice were used and all were at least 10 weeks of age.<br>All experiments were performed according to guidelines from the Canadian Council for Animal Care and under protocols approved by the Animal Care Committee of the Princess Margaret Cancer Centre, Toronto, Canada.
Wild animals	n/a
Field-collected samples	n/a

Ethics oversight	The Canadian Council for Animal Care and under protocols approved by the Animal Care Committee of the Princess Margaret Cancer Centre, Toronto, Canada

Note that full information on the approval of the study protocol must also be provided in the manuscript.

Flow Cytometry

Plots

Confirm that:

☒ The axis labels state the marker and fluorochrome used (e.g. CD4-FITC).

☒ The axis scales are clearly visible. Include numbers along axes only for bottom left plot of group (a 'group' is an analysis of identical markers).

☒ All plots are contour plots with outliers or pseudocolor plots.

☒ A numerical value for number of cells or percentage (with statistics) is provided.

Methodology

Sample preparation	Primary mouse mammary epithelial cells were obtained by collagenase and hyaluronidase exactly as described in Methods.
Instrument	Data was collected on a LSRII (Becton Dickinson), a FACSAria II (Becton Dickinson)
Software	Analysis was performed using FlowJo.
Cell population abundance	Epithelial cells consisted of approximately 30% of the total sample, and were considered abundant. For epithelial subpopulations, ~150ul of sorted samples were re-run to check purity and a population was considered pure if >95% of re-run cells fell within appropriate gates.
Gating strategy	A single cell suspension of primary mammary epithelial cells were gated on forward and side scatter area to select cellular events from debris. Next cellular events were gated as singlets, followed by negative selection gates to select live (DAPI negative) hematopoietic/endothelial depleted (CD31, CD45 and Ter119 negative) cells.<br>Next basal cells were gated as EpCAM low/CD49f+ and luminal cells were gated as EpCAM/CD49f positive. Hormone receptor negative luminal cells were gated as being CD49b+/Sca1- and hormone receptor positive luminal cells selected by CD49b-/Sca1+. Unstained/background, single positive and isotype staining controls were all used to determine positive gating.

☐ Tick this box to confirm that a figure exemplifying the gating strategy is provided in the Supplementary Information.
